# Metabolomics Based Study of the *Antileishmanial* Activity of Xanthium *strumarium* Leaf Extract on Promastigotes Phases of *Leishmania* major by Proton NMR Spectroscopy

**Published:** 2019

**Authors:** Mohammad AHMADI, Ziba AKBARI, Mahbobeh ALIKHANI, Reza HAJHOSSIANI, Zahra ZAMANI, Mohammad ARJMAND

**Affiliations:** 1. Metabolomics Laboratory, Department of Biochemistry, Pasteur Institute of Iran, Tehran, Iran; 2. Department of Biological Sciences, Payam Noor University, Tehran, Iran

**Keywords:** *Xanthium Strumarium*, *Leishmania major*, NMR spectroscopy, Metabolomics

## Abstract

**Background:**

*Xanthium strumarium* L. is extensively used as a traditional herb to treat many diseases and is also known as a source of phytochemicals. It has been used traditionally to treat trypanosomiasis, malaria fever, eczema, cancer, ulcer, fever, herpes headache, and skin lesion such as leishmaniasis. In this preliminary study, nuclear magnetic resonance (NMR)-metabolomics approaches was used to evaluate the inhibitory effects and metabolic alterations caused by leaf extract of *X. strumarium* on the stationary phases of promastigotes in *Leishmania major*.

**Methods:**

The promastigotes were cultured in Biochemistry Laboratory at Pasteur Institute of Iran in 2017, stationary phases were obtained from 5 to 6 day-old cultures and treated with different concentrations of the plant’s extract. Antileishmanial activity was assayed by MTT method and cell metabolites were extracted. ^1^H NMR spectroscopy was applied, and outliers were separated using multivariate statistical analysis.

**Results:**

The most affected metabolic pathways in the experimental groups were limited to amino sugar and nucleotide sugar metabolism, cyanoamino acid metabolism, starch and sucrose metabolism, butanoate metabolism, and galactose metabolism.

**Conclusion:**

The ethanolic leaf extract of *X. strumarium* is a potent growth inhibitor of *Leishmania major* and can affect vital metabolic pathways of Leishmania promastigotes. The assay provided new perspectives on the development of novel treatment strategies for leishmanial activity derived from natural products.

## Introduction

Leishmaniasis, the infection caused by several species of intracellular protozoa of the genus *Leishmania*, is identified as a neglected health problem by WHO, especially in tropical and subtropical regions. These infections exist in two clinical manifestations, visceral and cutaneous leishmaniasis (1). Cutaneous leishmaniasis (CL) is the most prevalent clinical form worldwide with 0.7 to 1.3 million new cases each year and annually causes 20,000 infection cases in Iran alone (1, 2). Since 1923, the chemotherapy based on pentavalent antimonials (SbV) has been the first-line treatment for leishmaniasis, which is far from satisfactory. Alternative drugs such as amphotericin B (amphoB), pentamidine, miltefosine, and drug combinations have also been recommended (3). The first indications of drug resistance were reported in the early 80s first in India and then in Nepal, in patients who had relapsed (4). The toxicity and the high cost of the available drugs due to drug resistance, teratogenicity and the need for the hospitalization of patients have resulted in the loss of drug effectiveness (5).

Therefore, due to the mentioned reasons, there is an urgent need to develop safer and more effective antileishmanial drugs with high efficacy. Folk medicine based on natural plants is used by approximately 80% of the world population. It contains about 25% of modern medicine, widely applied in the treatment of anti-parasitic diseases (6).

*Xanthium strumarium* L. (Cocklebur) is an annual weed, including 25 species belonging to the Asteraceae family, which grows in Iran between August and September with local common name, Tough or Zardineh *X. strumarium* is traditionally used to treat trypanosomiasis, malaria fever, eczema, cancer, ulcer, fever, herpes, headache and skin sores such as leishmaniasis (7). The species contain a class of plant terpenoids called Sesquiterpene lactones (STLs) which are classified based on their carboxylic skeletons, into xanthanolides and guaianolides. STLs are believed to be the active metabolites of *X. strumarium* and have caused considerable interest because of the broad range of their biological activities. Artemisinin for the treatment of malaria, xanthatin and xanthinosin for antitumor activity, parthenolide for treating migraine and thapsigargin for curing prostate cancer all belong to terpenoids classes (8).

*Leishmania* parasites undergo fluctuating environmental conditions in their life cycle; promastigotes proliferate in the mildly alkaline and glucose-rich environment of the midgut of sandfly and amastigotes grow in the acidic and glucose-poor environment of the phagolysosome of macrophages. There were significant changes in parasite metabolism during these developmental stages (9). The altered metabolic fluxes undoubtedly reflect adaptation to differing challenges that *Leishmania* naturally confronts in its two hosts. Glycolysis rather than gluconeogenesis is more important in promastigotes than in amastigotes. Differentiation of procyclic promastigotes to infective metacyclic promastigotes involves changes in membrane fluidity in lipophosphoglycan (LPG) and glycerolipids structures (10).

Antimonials have a complex multifactorial mode of action such as an inhibitory effect on trypanothione reductase of the parasite, and sodium stibogluconate [Sb(V)] inhibits fatty acid oxidation, glycolysis, and energy metabolism (11).

Metabonomics is one of the omics technologies, which is regarded as one of the functional responses of biological systems to pathophysiological stimuli or genetic modifications and is relevant to drug resistance studies (12). It is defined as a promising approach to study metabolic alterations within living samples after drug treatment (13). In fact, this approach directly links the metabolic profile of an organism to its corresponding genomic profile, which is widely used to find new pharmaceutical compounds (12).

Due to the lack of effective chemotherapy, the emergence of drug resistance and the growing interest in plant compounds for the treatment of parasitic diseases, this preliminary study aimed to explore the inhibitory effects of *X. strumarium* leaf extract and compare the metabolome fingerprint alterations on stationary phases of promastigotes in *L. major* treated with this substance.

## Materials and Methods

### Plant material

Leaves of *X. strumarium* were collected from August–September 2014 from around Kermanshah City, Kermanshah Province, Iran. Voucher specimens were authenticated by Central Herbarium of Tehran University (TUH) under voucher specimen number 48241.

### Preparation of crude plant extracts

The dried and powdered leaves of *X. strumarium* (40 g) were exhaustively macerated with an alcohol-water solution (80%) at room temperature for 72 hours. This mixture was filtrated and concentrated under reduced pressure to obtain a light-green extract. The resulting extract was mixed with active charcoal, centrifuged to isolate plant pigments and stored at −20 °C until required for bioassay.

### Determination of total polyphenol content

Total phenolic content was determined using the Folin-Ciocalteau reaction (2014). The calibration curve of gallic acid standard solutions was applied to calculate the number of total polyphenols. Measurements were done in tetraplicate (14).

### Leishmania parasites

Amastigote forms of *L. major* (strain MRHO/IR/75/ER) were originally isolated from infected Balb/c mice followed by transformation into promastigote forms in RPMI 1640 medium supplemented with 10–20% heat-inactivated fetal bovine serum(FBS), 100 μg/ml streptomycin, and 100 U/ml penicillin G at 23–26 °C (Sigma Aldrich, St. Louis, MO, USA). To perform leishmanicidal assays, Stationary-phase promastigotes were obtained from 5 to 6 day-old cultures and centrifuged at 3000 rpm for 10 min at 4 °C (15).

### Assay of leishmanicidal activity

The leishmanicidal assay was carried out following a protocol (16). The promastigote forms, in stationary phase, were seeded at 27°C for 24 h at 2×10^5^ promastigotes/well in a 96-well microplate. The stock solutions of each of the test samples (150 μg/mL) were added and serial dilution was carried out with RPMI medium. After 48 hours, promastigotes viability was calculated by tetrazolium-dye (MTT) colorimetric method and absorbance was measured at 545 nm in the presence of reference length wave 630 and the percentage of the viability of the promastigotes was measured according to IC_50_. The results were expressed according to the amount of the extract concentration (15–0.000015 μg/mL) required to reduce the absorbance to half (IC_50_) that of the negative control wells.

Amphotericin B(0.5 mg/ml) and promastigotes with no drugs were used as positive and negative controls, respectively

$$\text{Percentage}\;\text{of}\;\text{promastigotes}\;\text{viability}\;({\text{IC}}_{50})=\frac{\left(\text{Average}\;\text{absorbance}\;\text{in}\;\text{tetraplicate}\;\text{drug}\;\text{wells}-\text{average}\;\text{blank}\;\text{wells}\right)}{\left(\text{Average}\;\text{absorbance}\;\text{control}\;\text{wells}-\text{average}\;\text{blank}\;\text{wells}\right)}\times 100$$

### Cell extraction for metabolite analysis

Stationary promastigotes (initial concentration 1×10^9^ parasites/vial) were treated with IC_50_ concentrations of leaf extract (0.003 mg/ml^−1^ leaf extract/2×10^5^parasites). After incubation at 25 °C for 72 hours, the cells were centrifuged at 4000 rpm for 10 min at 4 °C. Then pellets were harvested for the extraction process of metabolites for ^1^HNMR spectroscopy. In brief, pellets (1×10^9^ promastigotes/vial) were resuspended in RPMI 1640 without FBS, centrifuged at 15000 rpm for 15 min at 4°C and washed twice with PBS. 1.0 ml of chilled perchloric acid (1.8 M) was added to the cell suspension, vortexed and sonicated for 5 min at 4°C followed by centrifugation at 12000 rpm at 4 °C for 10 min. The pH of the supernatant was adjusted to 6.8 and it was kept on ice for one hour to allow the precipitation of potassium perchlorate and then was centrifuged again as above. The supernatant was lyophilized (17).

### ^1^HNMR Spectroscopy

Before NMR acquisition, lyophilized powder samples (n=10) were resuspended in D_2_O containing trimethylsilyl propionate (1mM) as a chemical shift reference (δ=0 p.p.m.) and imidazole (2 mM) as pH indicator (δ=5.50 to 8.80 p.p.m.). Samples were centrifuged (18000 rpm for 30 min at 4 °C). 500 μl of the extracted samples were transferred to a NMR tube, analyzed on a Bruker AV-500 NMR spectrometer with field gradient operating at 500.13 MHz for proton observation at 298K. One-dimensional ^1^HNMR spectra were recorded using a 10-μs pulse, 0.1 s mixing time, 3.0 s relaxation delay, 6009.6 Hz spectra width, and 3000 transients with standard 1D NOESY pulse sequence to suppress the residual water peak (18).

### Data analysis

NMR spectra were preprocessed using custom written ProMatab function in MATLAB (v.7.8.0.347) environment. Following the standard processing steps, each 1D spectra were aligned and binned in 0.005 ppm and selected signals arising from water at 4.7 ppm were removed (19). Partial Least Square - Discriminant Analysis (PLS-DA), which is a supervised technique to visualize the class-specific segregation and to obtain the significant bins contributing to the variation across the classes, was used. This, in turn, led to the identification of the metabolites corresponding to the spectral bins using Human Metabolome Database (HMDB) and LeishCyc database, which is the pathway/genome database for *L. major*. Metabolic pathways were also investigated by the help of Metaboanalyst (v3.0) online software package.

## Results

### Total polyphenol content

The total phenolic content (TPC) of leaf extract of *X. strumarium* was equated to 0.150 μg/ml.

### Antileishmanial assay

*X. strumarium* stock extract (150 μg/mL) showed the highest anti-promastigote activity, while a weaker inhibitory activity against this parasite was observed in other concentrations. In comparison, amphotericin B presented an inhibitory activity almost similar to the stock fraction. Concerning the antileishmanial activity, we observed that only one concentration could be considered as having promising activity with an IC_50_ value of 0.15 μg/ml treating culture of stationary promastigotes (Table 1). There was a dose-dependent decrease in the percentage of viability.

**Table 1:** In vitro viability of *L. major* freidlin promastogotes against *Xanthium strumarium* extract activity

***Concentration(μg/ml)***	Promastigote viability (%) (Test-blank/negative control*100)	
*‘*	***Xanthium Strumarium***	***Amphotricin B (Positive control)***
Negative Control	85	80
150(Stock)	9	3
15	23	20
1.5	36	28
0.15	56	49
0.015	62	58
0.0015	76	76
0.00015	83	80
0.000015	82	79

### Effect of leaf extract of *X. strumarium* on metabolome profile

**Fig. 1:**
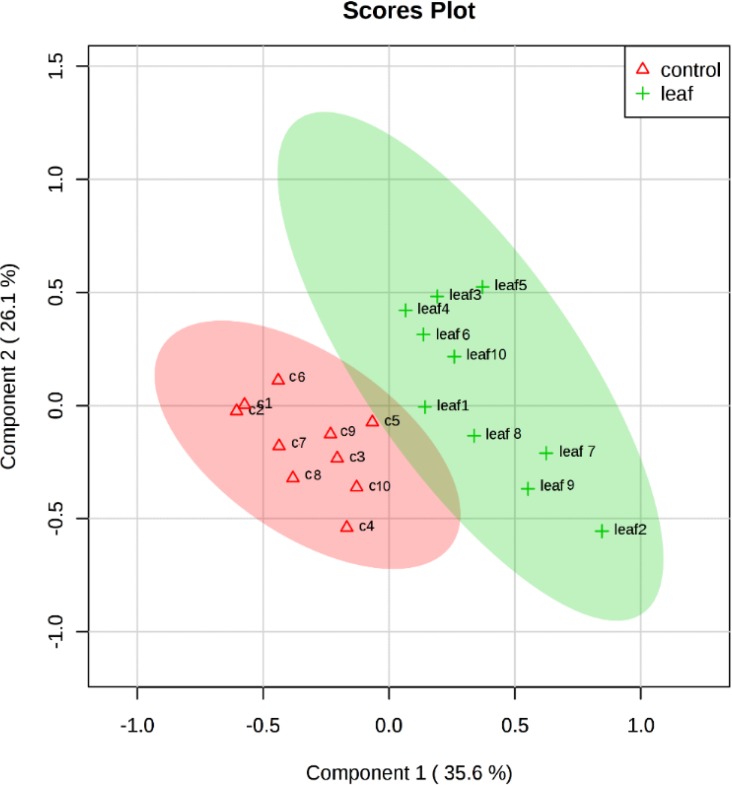
PLS-DA scores plot of stationary phases of *L. major* ^1^HNMR. One-color dots represent the sample available in a group. Red triangle = *L. major*–untreated and green cross = *L. major*-treated. The explained variance are shown in brackets

**Fig. 2:**
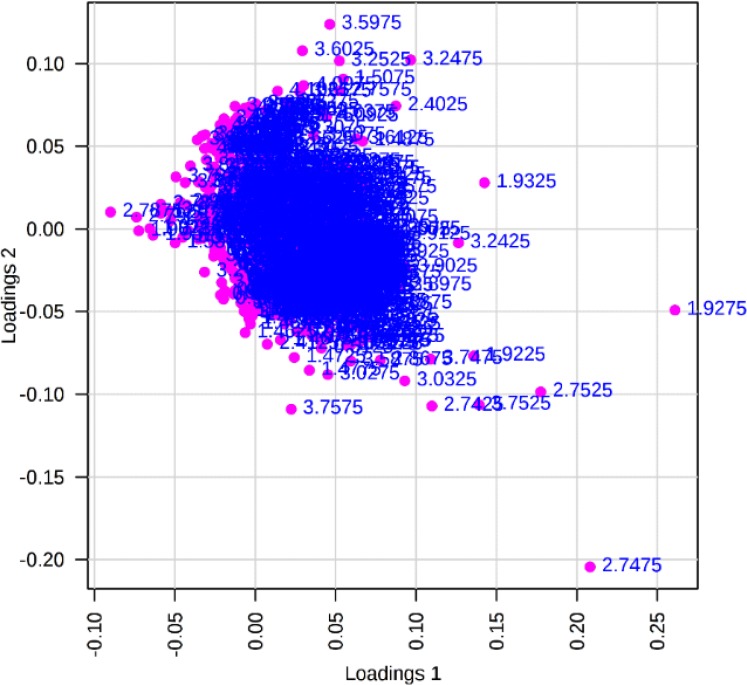
PLS-DA loading plot of stationary phases of *L. major*. This plot shows the relative contribution of bins/spectral variables to the clustering of experimental and control groups. Each point in the figure represents a bin. The loading [2] axis represents the correlation of the bin towards the predictive variation shown in Figure 1. The loading [1] axis represents the magnitude of the spectral bins

**Fig. 3:**
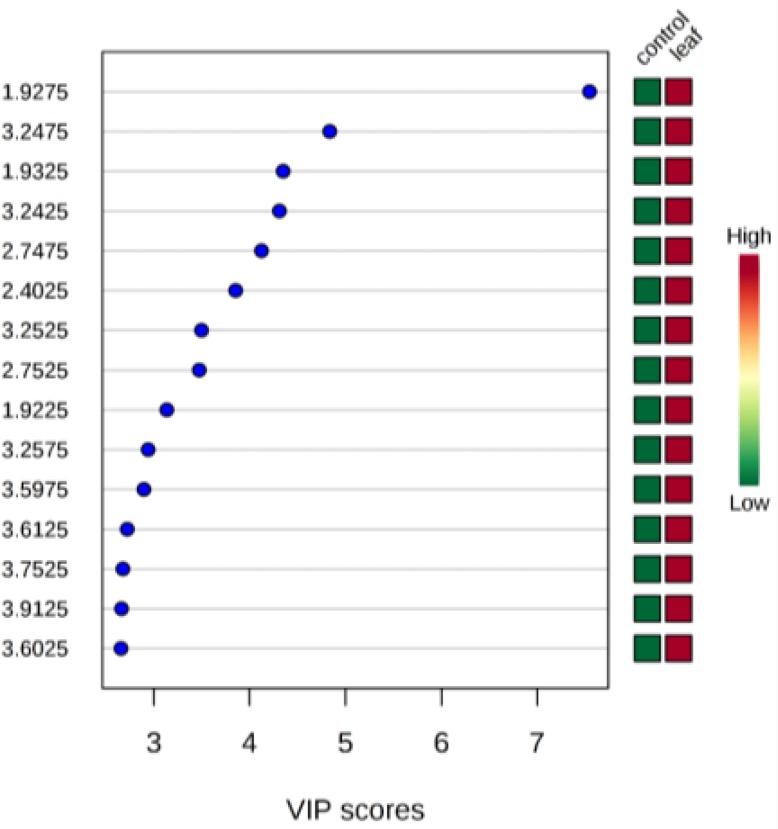
A VIP variable score is the measurement of the variable importance in PLS-DA model. The red color indicates the increase, and the green color stands for the decrease in variable concentrations

1D NMR assay showed that many metabolic pathways were influenced by leaf extract of *X. strumarium*. In the current study, the class-specific segregation can be refined using PLS-DA. This aided in the visualization of the data as scores plot (Fig. 1). Outliers were also separated by the use of loading plot (Fig. 2). Important features were identified by PLS-DA as variable importance of projection (VIP) (Fig. 3).

### Pathway analysis

**Fig. 4:**
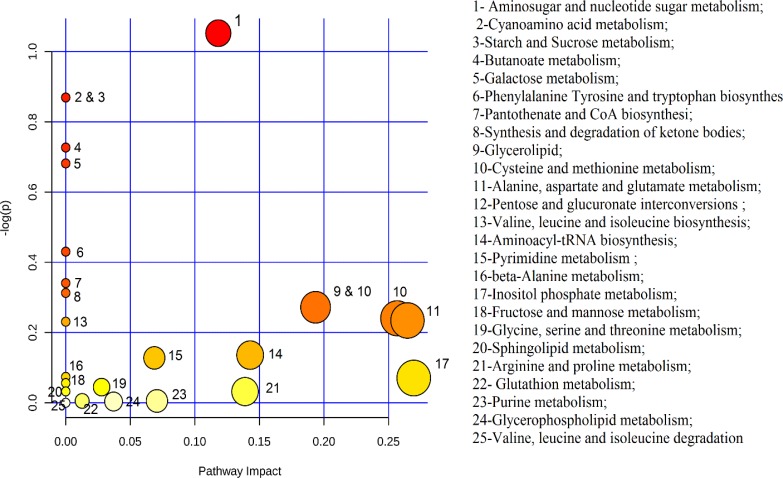
Topology map of altered biochemical pathways in experimental and control group according to the degree of centrality. The geometric position of each node in the topology map is presented by - Log(p)

Human Metabolome Database (HMDB) and LeishCyc were used to identify metabolites corresponding to the spectral bins. Also, metabolic pathways relating to these separated outliers were determined by generic databases, such as KEGG pathway database and Metaboanalyst online pathway analysis tools for metabolomics (Table 2 and Fig. 4). Five pathways with p value less than 0.5 were used in our discussion and other pathways were kept aside.

**Table 2:** Metabolome pathway analysis results

***Metabolic pathway***	***Metabolites***	***Total***	***Hits***	***Raw p***
Amino sugar and nucleotide sugar metabolism	D-Fructose, Glucosamine6-phosphate, uridine diphosphate galactose, Fructose 6- phosphate, N-Acetyl-D-Glucosamine6-phosphate	21	6	0.349
Cyanoamino acid metabolism	L-Aspartic acid, L-Asparagine	6	2	0.419
Starch and Sucrose metabolism	Glucose1-phosphate, D-Fructose	6	2	0.419
Butanoate metabolism	3-Hydroxybutyric acid, Butanol 1-Butanol	11	3	0.483
Galactose metabolism	Glucose1-phosphate, Uridine diphosphate galactose	7	2	0.506
Phenylalanine, tyrosine and tryptophan biosynthesis,	L-phenylalanine	4	1	0.650
Pantothenate and CoA biosynthesis,	Pantothenic acid L-valine	10	2	0.711
Synthesis and degradation of ketone bodies	3-Hydroxy butyric acid	5	1	0.731
Glycerolipid	D-glyceraldehyde	11	2	0.762
Cysteine and methionine metabolism	Cysteic acid S-Adenosyl homocysteine L-Serine L-Cystathionine	22	4	0.786
Alanine, aspartate and glutamate metabolism	Aspartic acid L-Asparagine glucosamine 6-phosphate	17	3	0.791
Pentose and glucuronate interconversions	glucose-1-phosphate	6	1	0.794
Valine, leucine and isoleucine biosynthesis	L-Valine	6	1	0.794
Aminoacyl-tRNA biosynthesis	L-Asparagine L-Histidine L-Phenylalanine L-Arginine L-Aspartic acid L-serine L-valine L-Lysine	46	8	0.873

Since, many pathways were tested at the same time; the statistical p values from enrichment analysis are further adjusted for multiple testing. Total is the total number of compounds in the pathway; Hits, the actually matched number from the user upload data; Raw p, the original *P* value calculated from the enrichment analysis by using Metaboanalyst (v.3) online software.

## Discussion

The toxicity profile of antimony justified the search for safer drugs in treating leishmaniasis. Antimony drugs inhibit energy metabolism and macromolecular biosynthesis via inhibition of glycolysis and fatty acid beta-oxidation (20). ^1^HNMR spectroscopy comparison of metabolome profile of *L. major* promastigotes treated with leaf extract of *X. strumarium* showed that there were important variations in some levels of individual metabolites and their corresponding pathways. The most significantly altered pathways between the two groups were amino sugar and nucleotide sugar metabolism, cyanoamino acid metabolism, starch and sucrose metabolism, butanoate metabolism, and galactose metabolism.

*Leishmania* parasites contain a variety of nucleotide sugars (21). One of the most distinctive roles of these nucleotide sugars is the biosynthesis of glycoconjugates, forming the cell surface coat (22). This dense glycocalyx is composed of glycosylphosphatidylinositol-like structures including lipophosphoglycans (LPGs), GPI-anchored glycoproteins, proteophosphoglycans(PPGs) and, free GPI glycolipids which are essential for parasite survival in the sandfly vector and the mammalian host (23).

The distinct role of sugar nucleotides in *Leishmania* molecular adjustment has been reported (22). *Leishmania* requires uridine diphosphate glucose (UDP-GLC) in the nucleus to synthesize an unusual DNA base called base J (β-D-glucosyl-hydroxymethyluracil) which plays an important role in regulating the onset and end of transcription (22). In the present study, treatment of *Leishmania* promastigotes with leaf extract of *X. strumarium* was associated with changes in N-acetylglucosamine 6-phosphate, glucose-1 phosphate, glucosamine-6 phosphate and glucose and fructose uridine diphosphate metabolites in amino sugar and nucleotides sugar metabolic pathways.

Collectively, it is implied that the plant extract, by affecting these metabolites, causes complex glycocalyx destruction, which in turn causes protective surface coat formation and mediates essential host-parasite interactions.

In the present study, the major alterations of amino acids in the cyanoamino metabolic pathway were confined to two metabolites, namely aspartic acid and asparagine. In another investigation, to find a novel drug, there were 97 unique metabolic reactions in *L. donovani*, which cyanoamino acid was one of them (24). Asparagine amino acid had an inhibitory effect on the autophagosome pathways of *Leishmania* (25). Increasing the amount of asparagine and decreasing the amount of aspartic acid by inhibiting asparaginase enzyme, could enhance the inhibitory effect of phagolysosome fusion and consequently inhibit promastigotes infection in the host body (26). In addition, the biological activity and the role of asparagine synthase enzyme are more emphasized today, due to the role of asparagine. Asp and Asn amino acids can be synthesized from oxaloacetate by mitochondrial enzymes of aspartate aminotransferase and asparagine synthase which were present in *L. major* (27).

There are two different A and B forms of asparagine synthase enzyme. Form A is strongly dependent on ammonium and form B uses glutamine. Form A of this enzyme is not observed in humans while it is considered a potential drug target in trypanosomes (28). Form A of this enzyme is necessary for the survival and infectivity of *L. donovani*. Therefore, it is considered a drug target (28). Previous studies revealed the important role of these two metabolites. Therefore, it is likely that *X. strumarium* leaf extract inhibits the growth of promastigotes by cyanoamino acid metabolic pathway alteration through increasing asparagine and reducing aspartic acid.

In addition, the present study indicates the alteration of two metabolites, fructose, and glucose 1 phosphate, in the pathway of starch and sucrose metabolism. Thus, sucrose is an intermediate metabolite in this metabolic pathway. The presence of a sucrase enzyme, namely fructofuranose, α-glucosidase, α-amylase, and cellulose in *L. major* was proven while it was absent in a few trypanosomes specie (29).

*L. major* promastigotes experience glucose-rich conditions in the digestive tract of their sandfly vector, as the sugar meals consumed from the plant sap were digested by amylase, kinase and sucrose enzymes (30).

In addition, the plant starch is hydrolyzed by α-amylase enzyme and converted to maltose, and maltose is often converted to glucose by α-glucosidase enzyme. Therefore, given that the main fuel of promastigotes is ATP production through glucose glycolysis, and since an important quantity of parasite glucose is provided through the pathway of starch and sucrose metabolism, it can be concluded that any change in the metabolites of this pathway can inhibit parasite growth by interfering with the energy of the promastigotes, which is consistent with our results.

In the current study, butanoate profile alterations are confined to three metabolites, 3-hydroxy-butyric acid and butanal and 1-butanol. No significant study was observed on the role and probable importance of butanol and butanal in the maintenance and survival of promastigotes. The previous metabolomics-based investigation, confirmed 3-hydroxybutyric alteration in mice treated with new amphotericin B drug compound (31).This drug compound had less toxic effects on patients with Leishmaniasis (32). Therefore, it can be postulated that butanol and butanal metabolites, especially 3-hydroxybutyric acid, play an important role in the parasite metabolism and the leaf extract of *X. stumarium* inhibits the growth of *Leishmania* promastigotes by disrupting this metabolic pathway and inhibiting ATP supply. Our results are supported by previous finding (31).

UDP-Gal can be interconverted to UDP-galactofuranose (UDP-Gal*f*) by the flavor dependent enzyme UDP-galactopyranose mutase (33). The LPGs were the most abundant glycoconjugates on the surface of *Leishmania* promastigotes, about 6×10^6^ copies per cell (34), which play an important role in protection against the hydrolytic enzymes of blood-fed phlebotomines (22). In *L. major*, The LPG galactose side chains are progressively capped with b1,2-linked D-arabinose during the transition from procyclic to metacyclic promastigotes. These changes lead to an increase in the thickness of the surface glycocalyx, which results in additional resistance to complement-mediated lysis (32).The defective galactose metabolism present in altered glycocalyx was associated with parasite attenuation (35).

Therefore, the importance of enzymes involved in the biosynthesis of Gal*f* might provide new targets for the development of effective drugs to combat Leishmaniasis (21). Deletion of the UGM gene in *L.major* resulted in the complete depletion of *β*-Galactofuranose (*β*-Gal*f*) in the structure of repeated units of phosphoglycans in GIPLs and LPGs. Furthermore, mice infection by this mutant of *L. major* was significantly attenuated (36). Moreover, galactose metabolism acts as a key factor in the occurrence of African sleepiness caused by *Trypanosome brucei* (37). In the present study, two key metabolites of glucose 1-phosphate and uridine diphosphate galactose in the galactose metabolic pathway changed in promastigotes treatment of promastigotes with leaf extract of *X. stramarium* caused disruption or change in the glycoprotein cover of the parasite, by disrupting or changing the galactose metabolism. Therefore, increased susceptibility to host complement and oxidative stress, caused promastigotes attenuation.

## Conclusion

Ethanolic leaf extract of *X. stramarium* exhibits antileishmanial activity even in low doses and affects vital metabolic pathways of *Leishmania* promastigotes. Further research is underway to find out the potential fractions of the leaf extract of this plant.
